# Investigating the role of bulking agents in compost maturity

**DOI:** 10.1038/s41598-023-41891-y

**Published:** 2023-09-25

**Authors:** Khadija Zahra, Muhammad Farhan, Amina Kanwal, Faiza Sharif, Muhammad Umar Hayyat, Laila Shahzad, Gul Zareen Ghafoor

**Affiliations:** 1 Sustainable Development Study Center, Government College University, Lahore, Pakistan; 2 Department of Botany, Government College Women University Sialkot, Sialkot, Pakistan

**Keywords:** Environmental monitoring, Pollution remediation

## Abstract

Kitchen waste is increasing globally, similarly in Pakistan bulk of municipal solid waste comprises of kitchen waste specifically, tea waste. Composting of kitchen waste is one of the promising ways to convert waste into useful product, resulting into zero waste. This study is aimed to convert waste (kitchen waste) in to a resource (compost) using bulking agents (tea waste and biochar) for reducing maturity time. Secondly, compost application on *Solanum lycopersicum* (tomato) was also tested. Four compost treatments were designed under aerobic composting conditions for 30 days. Tea waste and biochar have accelerated the maturity rate and produced a nutrient rich compost. Final compost had Electrical Conductivity of 2mS/cm, Carbon Nitrogen ration of 15, 54% of organic matter, 15% of moisture content, 48% of cellulose content, and 28% of Lignin content. With the use of Co-compost the *Solanum lycopersicum* showed 133% germination index, 100% germination, 235% Munoo-Liisa Vitality Index and 1238% seed vigor index. Co-compost also improved the soil total nitrogen by 1.4%, total phosphorous by 2%, total potassium by 2.1% and bulk density by 2.6 gcm^−3^. This study successfully used tea waste and biochar as bulking agents to reduce maturation time to 30 days. Tea waste and biochar enhanced the organic matter degradation, lignocellulose degradation, water holding capacity, porosity, seed’s vigor, germination index. This research can be helpful in developing home composting and home gardening to combat solid waste management and food security issue in developing countries.

## Introduction

The unprecedented population growth is a root cause for a lot of environmental problems. Organic waste production at household level varies in different locations as per the lifestyle and economic conditions. For instance, organic waste production in developing countries like India, Nigeria and Jordan is 45–80%. In Pakistan the annual increase in solid waste generation is 2.4% and an average person produces 0.6 kg of waste daily. The generation from one household ranges from 1.9 to 4.3 kg/day in major cities of Pakistan^1^. Solid waste is generally divided into farm waste, municipal waste, kitchen waste, hospital waste, building waste, and plastic waste^2^. Kitchen or household waste is the most neglected/non-recycled part of the waste and its global production is approximately 1.2 Bt per annum^3^. Kitchen waste (KW) has a very high potential to contribute in economy and environmental well-being but only if it is managed properly. It can cause many direct or indirect impacts, where direct impacts include emissions of greenhouse gases (GHGs) and public health deterioration. Kitchen waste is responsible for 8% of overall GHGs emissions worldwide^4^. While as an indirect impact, the recyclable waste is either gets incinerated or dumped^5^. Due to COVID-19 the fluctuation in generation of household waste has been recorded. This change varies from one country to another due to economic, cultural, ethical, mental and demographic factors^6^.

Developing countries like Pakistan, Nepal, Bangladesh, India etc. follows the same old practices of kitchen waste management that generally includes incineration, combustion, open dumping or landfills. Composting is one very efficient and underutilized way of employing the true potential of kitchen waste. Kitchen waste contains very high quantities of essential nutrients like NPK and bioavailable organic matter (OM) which supports the microbial, chemical and physical activities of composting process. If correctly converted into compost the toxic waste can be converted into an extremely valued product, both in economic and environmental terms^7^. During composting, the waste goes through a process of mineralization to convert binded elements into inorganic soluble forms. Consequently, when the compost is applied to the soils the nutrients are in bioavailable form and the plants can easily take them up^8^.

Studies suggest that compost application brings out positive results and improves the fertility of soils^9^. Traditional composting technique is time taking and it also has less available nutrients in final compost because of high carbon and nitrogen loss during the long periods of composting. Unlike traditional composting, two stage composting is quite a new technique this help in shortening the time period for making compost by extending the thermophilic stage of compost by adding different bulking agents^10^. The common problems during kitchen waste composting are that KW is rich in cellulose and lignin, have high moisture and little C/N ratios, these problems are tackled with addition of bulking agents^11^. The interest has been increasing about studying the impacts of biochar on soil and plants. Despite all the positive impacts of biochar it has been noted that direct applications of biochar has the tendency to immobilize available nitrogen^12^ which ultimately will cease plant growth^13^. This happens because biochar has a rich supply of carbon which in some circumstances, dominate the available soil nitrogen. If biochar is co-composted with tea waste, then the composting process will accelerate and the final compost will be very rich in nutrients^2^.

The major benefits of using bulking agent for compost are increased aerobic conditions and high surface area which support microbial activity^14^. Bulking agents also helps to extend thermophilic phase by increasing enhance temperature^15^. Another promising benefit is that with addition of bulking agents the emission of gasses is reduced, this is very useful in controlling odor and greenhouse gas potential^11^. Nutrient retention in soil is improved and results in more fertility^16^. The raw materials used as bulking agents include, dry leaves, tree pruning^2^, maize stalk^17^, bean dregs^3^, garden prune, saw dust^4^, wood chips^18^, cornstalk^5^, wheat straw^6^, rice husk^7^. Compost maturity is one important factor that needs to be assessed properly because immature composts can be very harmful for plants, soils and environment^19^. However, the impact of biochar and tea waste as bulking agents, in quality improvement and maturity of kitchen waste composting has not been widely reported. The key objectives of this study are to convert kitchen waste into a compost using bulking agents. Secondly, we tested the effectiveness of prepared compost on plant growth.

## Materials and methods

### Raw materials for compost

Kitchen waste (KW) was collected from three canteens in Government College University, Lahore (GCU) and comprised of materials like fruit peels, vegetable peels, eggshells, bones, plastic materials, and glass. The waste was then segregated manually to remove non-degradable materials (plastic, glass, etc.)^7^. Kitchen waste was then crushed in a grinder to reduce the size and increase surface area^8^. Tea waste (TW) which is residue that remains after cooking tea, was collected from local Café situated at Barkat market, Lahore. TW and KW samples were air dried for 3 days without exposing the samples to direct sunlight. Biochar (BC) was made from wheat husk which was collected from a local flour mill, and it was then combusted at 550 °C in muffle furnace under controlled conditions, in Soil and Plant Lab of Sustainable Development Study Centre, GCU Lahore.

### Composting process and sampling

The process was conducted at household level for the duration of 30 days from July 2022 to August 2022. Aerobic indoor composting was chosen for this study^13^. Compost pile was made in pot with a dimensions of 30 × 30 cm (length x diameter). Layers were established to make a compost. KW and BAs were added in 3:1 weight ratio, respectively^20^. 10 kg compost pile was created for each treatment. To maintain the moisture of the compost pile 10 ml water was added after every 6 days for initial 12 days. Compost pile was turned every day after noting temperature to maintain proper conditions for composting^21^. Samples were taken on days 2, 10, 20, and 30. Samples were collected each time from three depths (near the surface of pile, midway of pile, and core of the pile) and they were mixed to create a composite sample, samples were then placed in a clean plastic bag at 4 °C to perform further tests^17^. Four treatments were designed for this study with three replicates (Table 1).

**Table 1:** Compost treatments and their composition.

Treatments	Composition	Replicates
T1	Kitchen waste (Control group)	T1R1, T1R2, T1R3
T2	Kitchen waste + Tea waste	T2R1, T2R2, T1R3
T3	Kitchen waste + Biochar	T3R1, T3R2, T1R3
T4	Kitchen waste + tea waste + Biochar	T4R1, T4R2, T1R3

### Physical and chemical analysis

Electrical conductivity (EC) and pH was determined in a 1:10 (w/v) water soluble extract^4^. Moisture content (MC) was measured by dyring the compost samples at 105 °C till constant weight is achieved^21^. Organic matter (OM) was quantified by loss on ignition (LOI) method in muffle furnace (550 °C)^22^. Total carbon (TC) and total nitrogen (TN) was assessed by automatic elemental analyzer^17^. Cellulose and lignin contents are calculated using reflux method of Keeflee et al.^23^ using Eqs. (1), (2), (3), and (4).

1 $$Cellulose\, content=weight\, of\, sample-weight \,of\, Ash,$$

2 $$\% \,Cellulose=cellulose\, content\, \times\, 100,$$

3 $$Lignin\, content=weight\, of\, sample-weight\, of\, Ash,$$

4 $$\% Lignin=Lignin \,content \times\,  100.$$

Water holding capacity (WHC) and bulk density was calculated using Eqs. (5) and (6) as reported by Ahn et al.^20^;

5 $${\mathrm{Water \,holding \,capacity }}=\frac{[\left(Ms-M1\right) + MC \times\,  M1]}{[\left(1-MC\right)\times\,  M1]},$$

where Ms = weight of sample after filtration, M1 = weight of initial sample, MC = moisture content of initial sample.

6 $$Bulk \,density (g/{cm}^{3}=\frac{\mathrm{weight\, of \,the\, sample }(\mathrm{g})}{\mathrm{volume \,of \,sample }(\mathrm{cm}^3)}$$

Porosity was calculated according to Tautmann and Krasny^24^ using Eq. (7) given below;

7 $$\mathrm{Porosity}=\frac{\mathrm{PSV}}{\mathrm{Vi \times\,  }100}$$

where PSV = pore space volume, Vi = volume of initial sample.

Germination index GI was calculated according to Abdel and Al-Shaieny^25^ using Eq. (8);

8 $$\mathrm{Germination \,index }(\mathrm{GI})=\frac{\sum \mathrm{Gs}}{\mathrm{D}}$$

where Gs = total number of germinated seeds, D = number of days.

### Pot experiment

The seeds of tomato were soaked in warm distilled water for 3 h. Pots (30 × 30 cm, length × width) were filled with soil and successive treatment of compost. The pot treatmnets are shown in Table 2. Seeds were then sown into each pot and the experiment lasted for 30 days. Each pot was sprayed with sufficient amount of water daily to maintain the optimal moisture conditions^6^. Growth was measured by counting number of leaves and height of stem. To measure height of stem measuring rod was placed at the bottom and was taken to the top of the stem. Leaf length was measured from the point where the leaf joins the stalk to the pointy end of the leaf^8^. Leaf width was measured from 1 end to the other at the widest part of the leaf^9^. All the readings were taken in triplicates.

**Table 2:** Treatments for pot experiment, ( +) shows addition while (−) shows no addition.

Treatment	Soil	Compost type				Replicates
		T1	T2	T3	T4	
Po	+	−	−	−	−	PoR1, PoR2, PoR3
P1	+	+	−	−	−	P1R1, P1R2, P1R3
P2	+	−	+	−	−	P2R1, P2R2, P2R3
P3	+	−	−	+	−	P3R1, P3R2, P3R3
P4	+	−	−	−	+	P4R1, P4R2, P4R3

### Statistical analysis

All the data is presented in mean values of three samples. The data was analyzed using Microsoft Excel 2016 (Microsoft Corporation, USA) and SPSS Statistics 16.0 software. Post Hoc test, Tuckey test, descriptive analysis, least significant difference LSD, One-way and Two-way ANOVAs were performed^26^.

## Results and discussion

### Temperature, pH and electrical conductivity changes during composting

**Figure 1:**
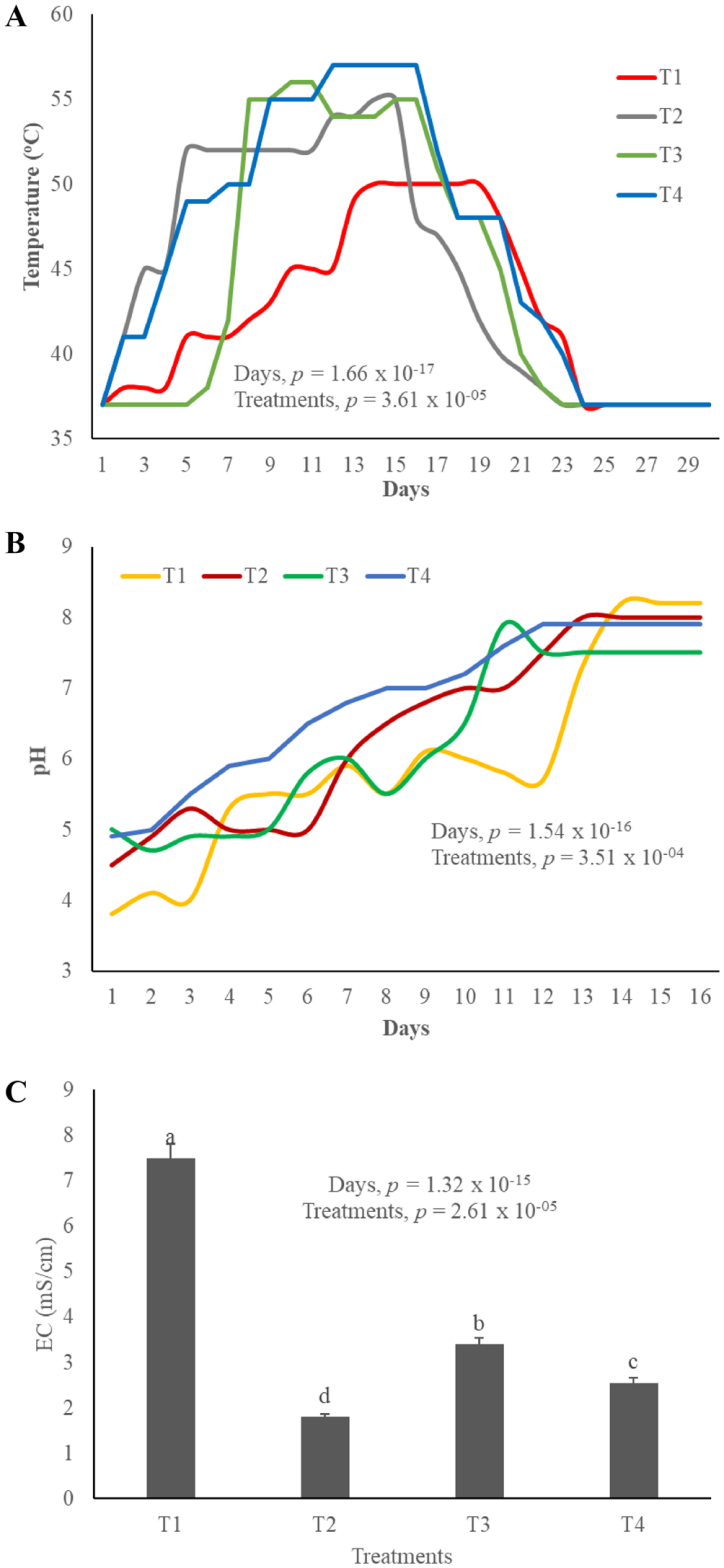
Changes in parameters during composting process, (**A**) Temperature changes, (**B**) pH changes and (**C**) Electrical conductivity.

Temperature is greatly influenced by different treatments *p* < 0.05. Temperature rise in T_3_ is very abrupt compared to the other treatments. While the shift in T_4_ from thermophilic phase to cooling phase is quite sharp. T_2_ showed more gradual temperature change than the other treatments. All the three treatments except T_1_ have maintained a very high temperature (50–60 °C) for more than a week (Fig. 1A). T_3_ and T_4_ showed sharp temperature changes, this might have happened because of the presence of biochar, as it has a great water absorption potential which cause the rapid changes in temperature^27^. High temperature is achieved by the activities of microbes^28^, this rapid microbial growth is facilitated by high surface area of biochar and tea waste^29^. T_1_ (having no bulking agent) did not achieved high and long thermophilic phase, which was essential to kill pathogens^30^. Biochar has good amount of OM available which also enhances the microbial and enzymatic activity^31^, resultantly the temperature rises^32^. During the composting period pH vary between 4 and 8 because of the different reactions happening in the compost pile^33^. Different studies have established that pH drops at the start of composting^24^ because of the development of anaerobic conditions^25^ and consequently organic acids formations^34^. It is evident in that pH values are also influenced by different treatments *p* < 0.05. T_4_ gradually moves from acidic to alkaline pH while T_3_ again shows abrupt changes in pH. On the other hand, pH in T_2_ turns acidic in the mesophilic phase and then rise to alkaline gradually (Fig. 1B). In T_1_, pH remained acidic for a very long time even in the thermophilic phase, this might be because of the high organic acid concentration^31^, low mineralization^17^ and slow degradation rates of OM/organic N^35^. While in other three treatments pH started to move towards neutral as the thermophilic stage approached. This is because of the aerobic conditions, high temperatures^31^, mineralization of organic nitrogen^17^ and degradation of organic matter^36^. EC of the compost represents the salt content of the mature compost^37^. It is an important parameter in terms of explaining the effect of compost on plant growth^38^. EC is directly linked to the pH values as the pH increases EC also increases^35^. EC values of the three treatments T_2_-T_4_ are under safe EC range of 0-4mS/cm, while T_1_ (no bulking agent) has EC value higher than 4 mScm^-1^ (Fig. 1C). High EC values present the threat of salinity in the soil which can be harmful or even fatal for the plant growth^25^. High EC range is because biochar release high amounts of soluble ions and have higher potential of accumulating different ions^11^ due to great surface area^39^.

### Moisture content, water holding capacity (WHC) and porosity changes during composting

**Figure 2:**
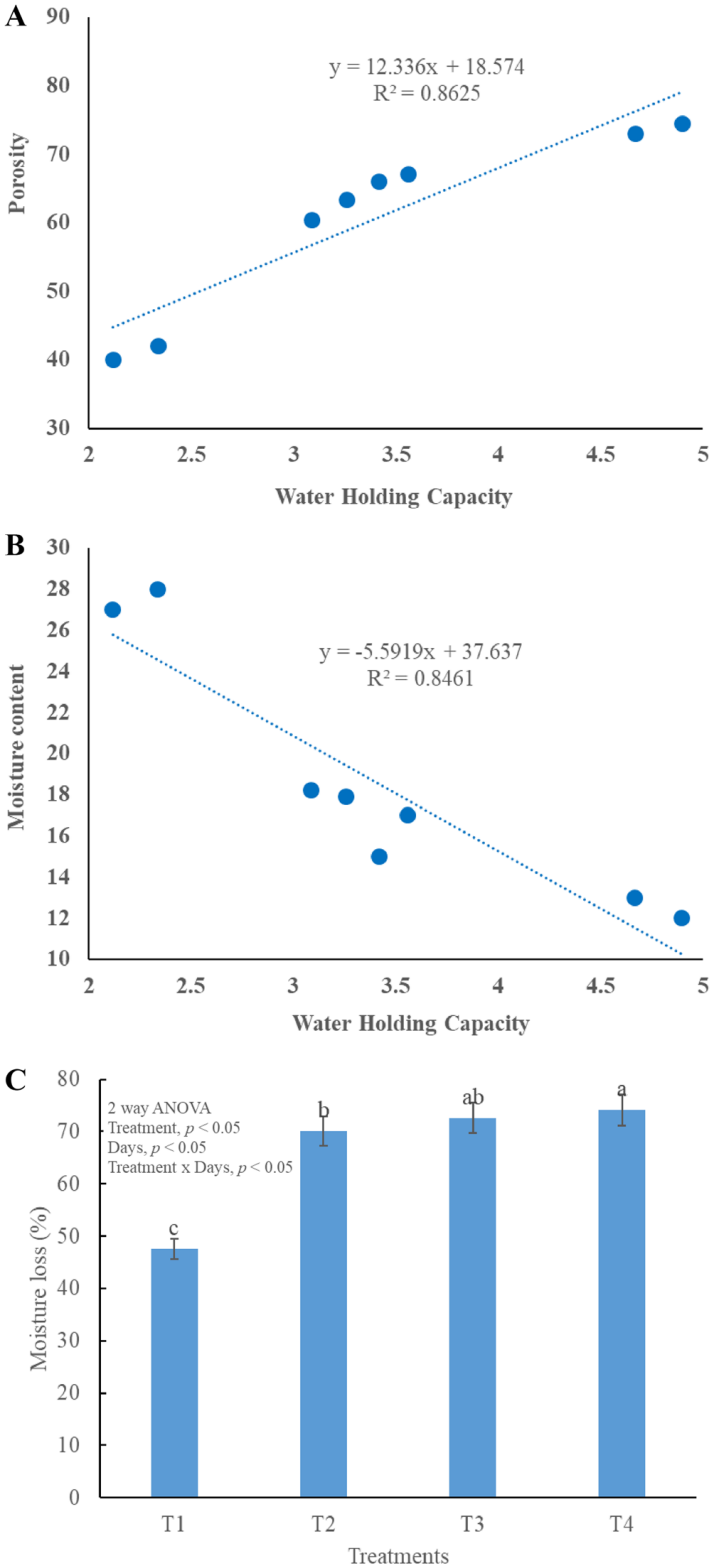
Correlation between different variables, (**A**) Water holding capacity and porosity, (**B**) Water holding capacity and moisture content, (**C**) moisture loss in different treatments.

Moisture content determines the fate of composting and nutrient content of the compost. Higher MC result in nutrient leaching and it does not allow the temperature to rise. Moisture content of all the treatments was remained in the optimum range, except T_1_ (Fig. 2A–C, Table 3). At the start of composting process MC should be in 50–65% range to stop the nutrient loss from leaching^40^. At the end of composting MC must be in 15–25% range and this criterion is not followed by T_1._ MC has an indirect impact on the WHC and porosity^41^ of final compost (Fig. 2). As MC increases porosity and WHC start to decrease^42^. WHC is significantly different is all treatments *p* < 0.05. T_4_ shows the highest water holding capacity. WHC of compost should be high so that the nutrient loss can be reduced, aggregate size and soil structure can be enhanced^27^. In this study, all treatments WHC are significantly different (*p* < 0.05) from each other. WHC of T_1_ is very low while T_4_ has highest WHC, this is due to the higher surface area and adsorption capacity of bulking agents^3^. Porosity is also an important parameter which influence soil quality and plant growth. This is also dependent upon the moisture^21^ content and bulk densities^20^. Optimum conditions of porosity and WHC are directly proportional to the type and quantity of bulking agent^42^.

**Table 3:** Maturity indices of final compost.

Parameters	T1	T2	T3	T4
TN%	0.77 ± 0.02	1.24 ± 0.04	1.23 ± 0.02	1.34 ± 0.04
TP%	0.24 ± 0.01	1.12 ± 0.02	1.36 ± 0.04	1.89 ± 0.02
TK%	0.31 ± 0.01	1.49 ± 0.03	1.91 ± 0.04	2.06 ± 0.02
Moisture content%	32.20 ± 0.64	18.11 ± 0.36	16.2 ± 0.49	15.13 ± 0.15
WHC g wet/g dry sample	2.23 ± 1.21	3.17 ± 0.10	3.49 ± 0.07	4.78 ± 0.14
Porosity %	40.20 ± 0.0	61.82 ± 1.24	69.40 ± 2.08	73.71 ± 0.74
Root length (cm)	0.55 ± 0.01	0.65 ± 0.01	0.73 ± 0.01	0.97 ± 0.01
Shoot length (cm)	3.05 ± 0.06	6.84 ± 0.14	7.31 ± 0.22	11.40 ± 0.11
Stem height (cm)	5.80 ± 0.17	14.01 ± 0.28	14.50 ± 0.44	18.25 ± 0.18
No. of leaves (cm)	8.00 ± 0.08	28.00 ± 0.56	30.00 ± 0.60	37.00 ± 0.74

### Organic matter content and C/N ratio variations in compost

**Figure 3:**
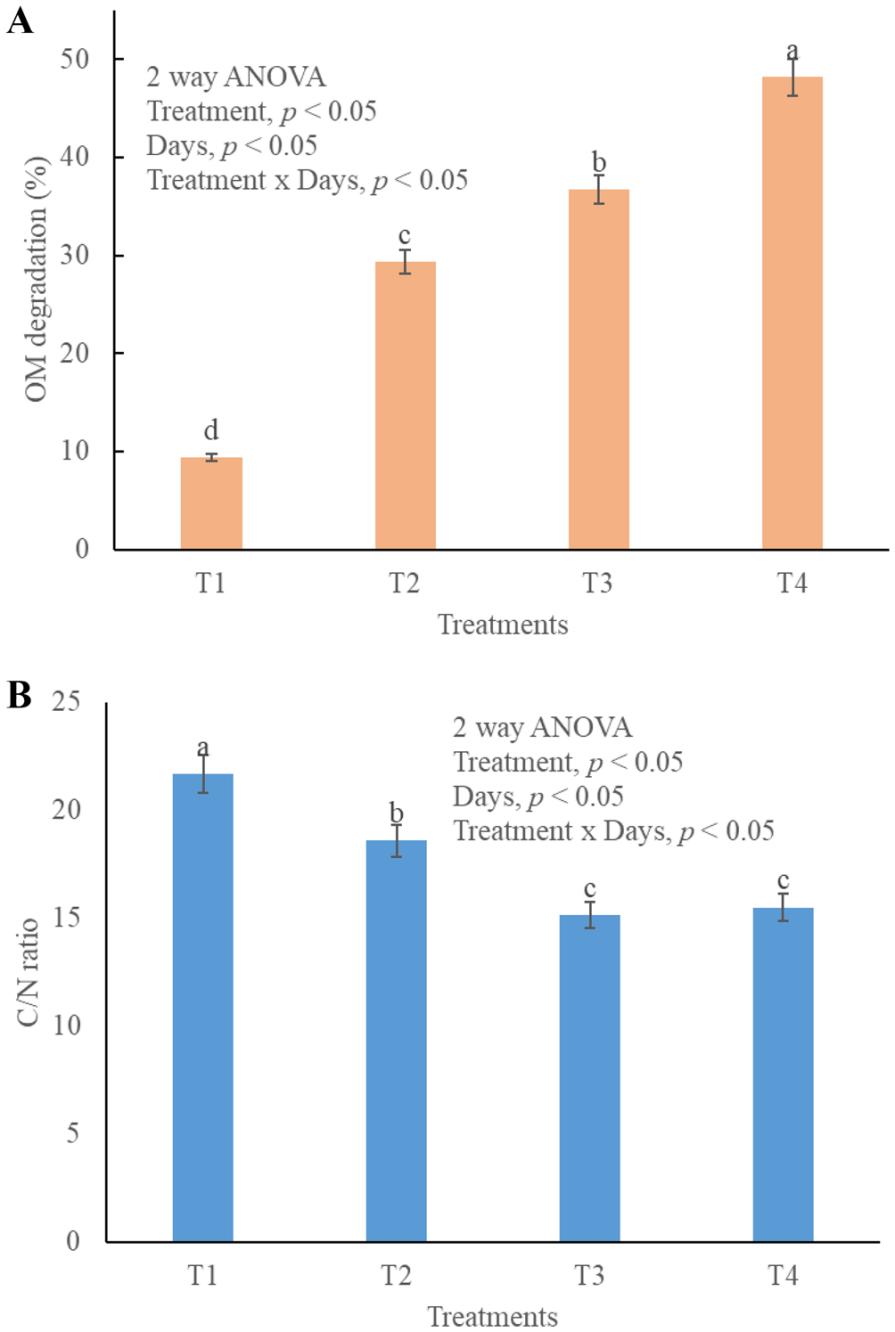
Final ratios in different composting treatments, T1 = kitchen waste, T2 = Kitchen water + tea waste, T3 = Kitchen waste + biochar, T4 = Kitchen waste + tea waste + biochar, (**A**) Organic matter degradation in different treatmnets, (**B**) C/N ratio in different treatments.

OM is one very essential parameter to determine the maturity of compost. Different treatments are significantly different (*p* < 0.05) for OM (Fig. 3A). T_4_ showed the highest degradation rate and high OM at the start of composting process. T_2_ and T_3_ showed comparatively slow degradation rates (Fig. 3A) and it declined with the passage of time. T_1_ is considered immature compost because the OM content was only 16% and to be a mature compost, the OM content should be around 30–50%. OM content keep on decreasing as it is used by microbes as energy^43^ and food source^35^. Organic C is also lost during composting through volatilization in form of CO_2_^44^. Low degradation rates are low in T_1_ because there is no bulking agent to support the growth of microbes^45^. TW and BC both promoted OM degradation due to their large surface area for the nurturing of microbes^6^. High adsorption capacity of bulking agents provide sufficient nutrients for microbial community^46^. Biochar promotes the enzymatic activities and is able to decompose even the insoluble OM in the compost pile^47^. Optimum C/N is also very important parameter for successful composting and is a potential indicator of compost maturity^48^. Different compost treatments *p* < 0.05 are significantly different from each other in C/N ratio. The C/N ratio of T_1_ is higher than 18 and this can cause excessive mineralization or immobilization of N, which is not beneficial for the plant growth. All the other three treatments (T_2_, T_3_ and T_4_) are under 14–18 range which is indicative that compost is matured and can be used to enhance plant growth (Fig. 3B).

In order to determine the carbon and nitrogen content of the compost the ratio of waste to bulking agent should be adjusted^40^. Appropriate C/N ratio is vital to sustain the microbial population in the most active form to accelerate composting process^49^. High C/N ratio can cause delay in maturation period while lower C/N can cause rapid N losses through volatilization or runoff. C/N ratio of final compost should be 14–18 which ensures slow mineralization of N when applied to the soil^48^.

### Cellulose and lignin degradation

**Figure 4:**
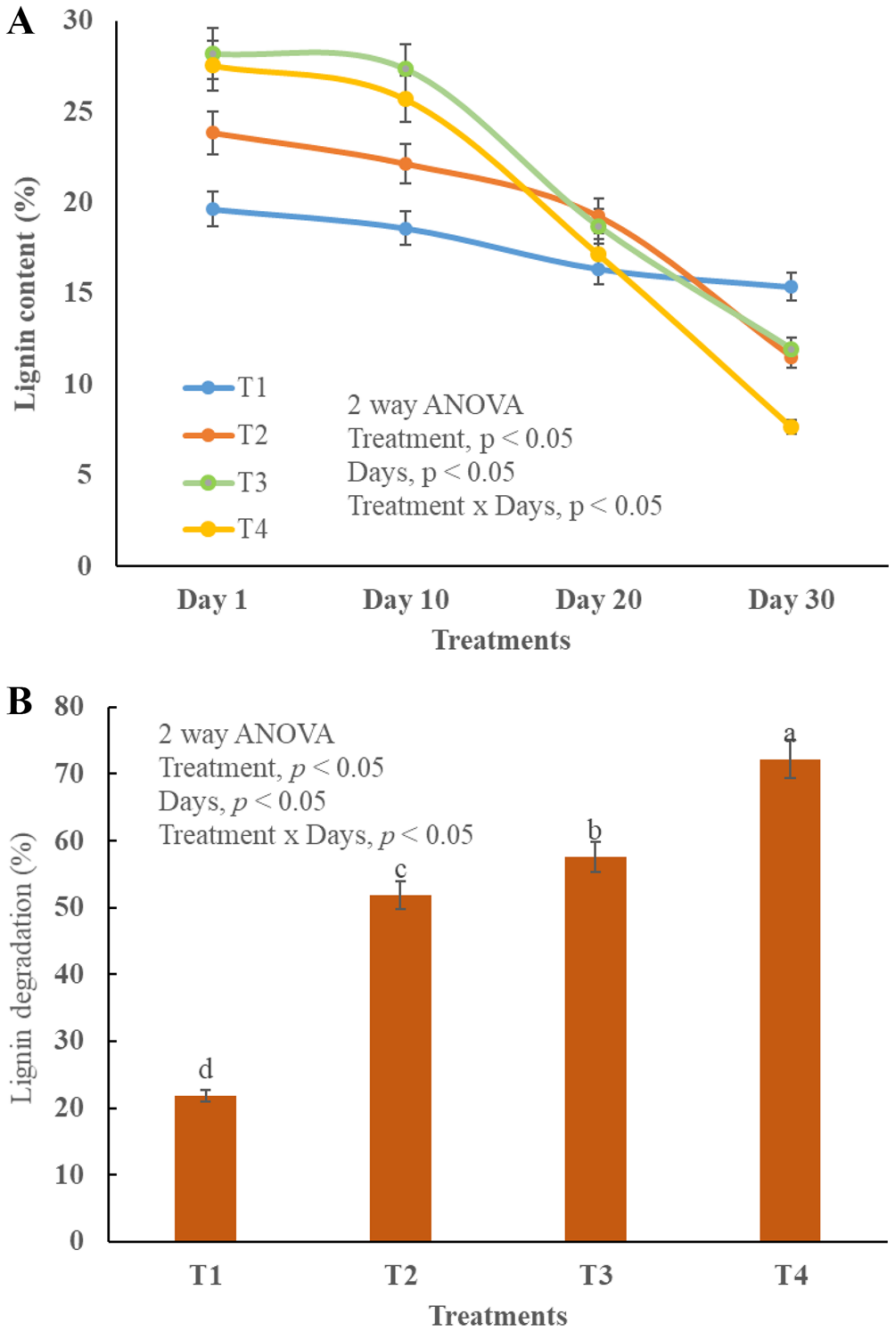
Changes in lignin during composting in different treatments, T1 = kitchen waste, T2 = Kitchen water + tea waste, T3 = Kitchen waste + biochar, T4 = Kitchen waste + tea waste + biochar Lignin degradation, (**A**) Changes Lignin content, (**B**) Changes in lignin degradation.

**Figure 5:**
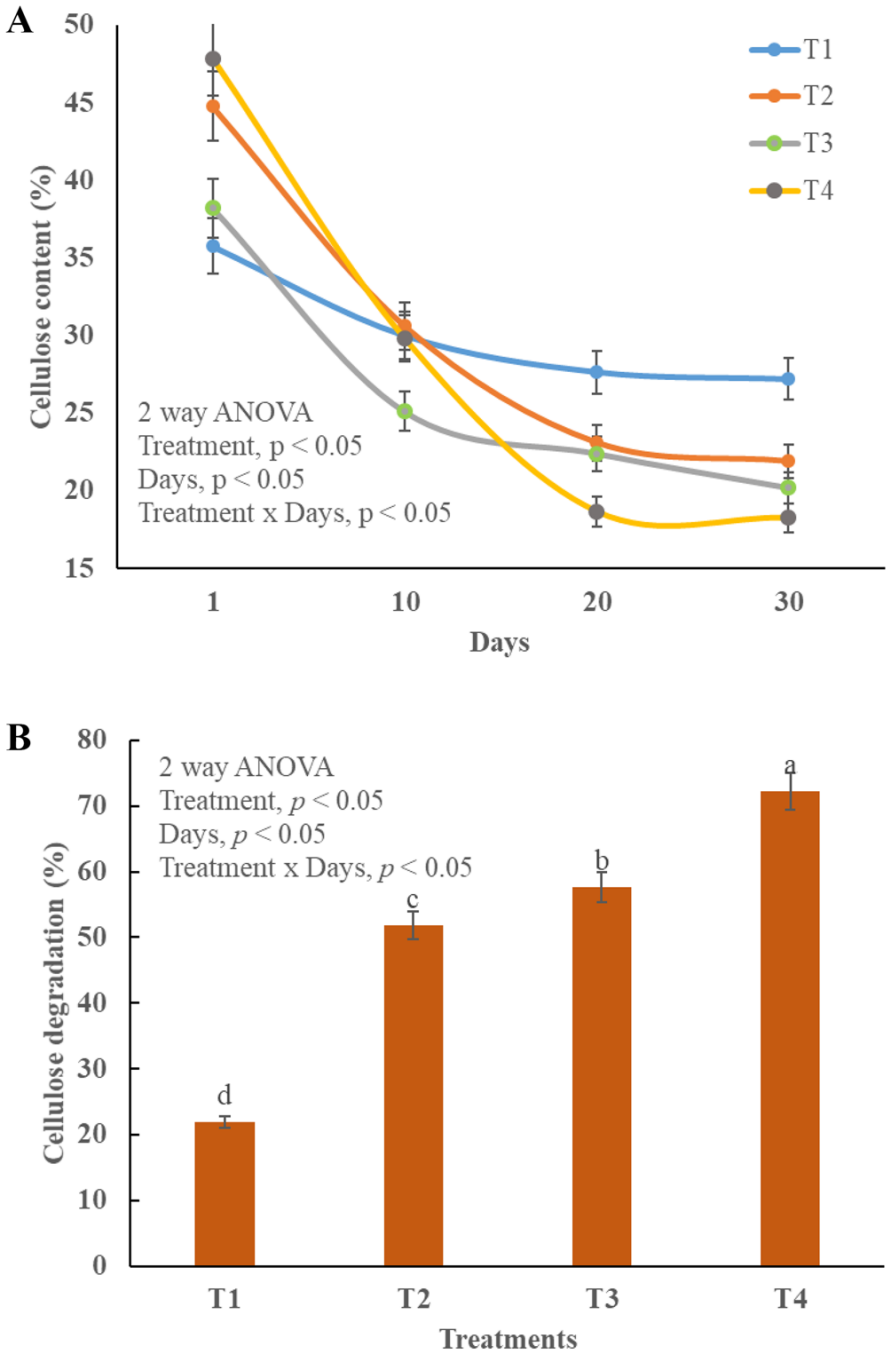
Changes in cellulose during composting in different treatments, T1 = kitchen waste, T2 = Kitchen water + tea waste, T3 = Kitchen waste + biochar, T4 = Kitchen waste + tea waste + biochar Lignin degradation, (**A**) Changes in cellulose content, (**B**) Changes in cellulose degradation**.**

Lignin is mostly degraded by mesophilic bacteria in presence of less OM, thus it is degraded mostly in cooling phase (Fig. 4A,B). Statistical analysis revealed that all the treatments are significantly different (*p* < 0.05) from each other (Fig. 4A,B). T_4_ showed excessive degradation (28–9%) potential because BC and TW offered high surface functional groups and optimal conditions for enzymatic activities^50^. Similarly, the absorption and strength of enzymes increased due to microbial abundance in BC and TW^51^. Cellulose content was high in the start of the experiment and at gradually decrease with the passage of time (Fig. 5A,B). The maximum degradation (73%) was observed in T_4_ and all the treatments differ significantly different (*p* < 0.05) Cellulose degradation is vital for both compost maturity and bioavailable of nutrients for plant growth^52^.

### Micronutrients in compost

NPK is an essential parameter in compost maturation as this increase the plant growth. In present study, all four treatments have different nutritional value (*p* < 0.05) (Table 2). T_4_ have shown enough NPK while T_1_ did not meet the target. T_2_ and T_3_ have almost the same amounts of NPK. These results are in agreement with the pot experiment where stem height and number of leaves were increased in all three treatments compared to the control, while T_1_ showed slightly lower stem height than control treatment. Higher ratios of NPK can also cause immobility of the nutrients into the roots of plants^11^. Lower NPK ratios also can cause nutrient deficiency in the plants^47^.

### Germination index and germination percentage

**Figure 6:**
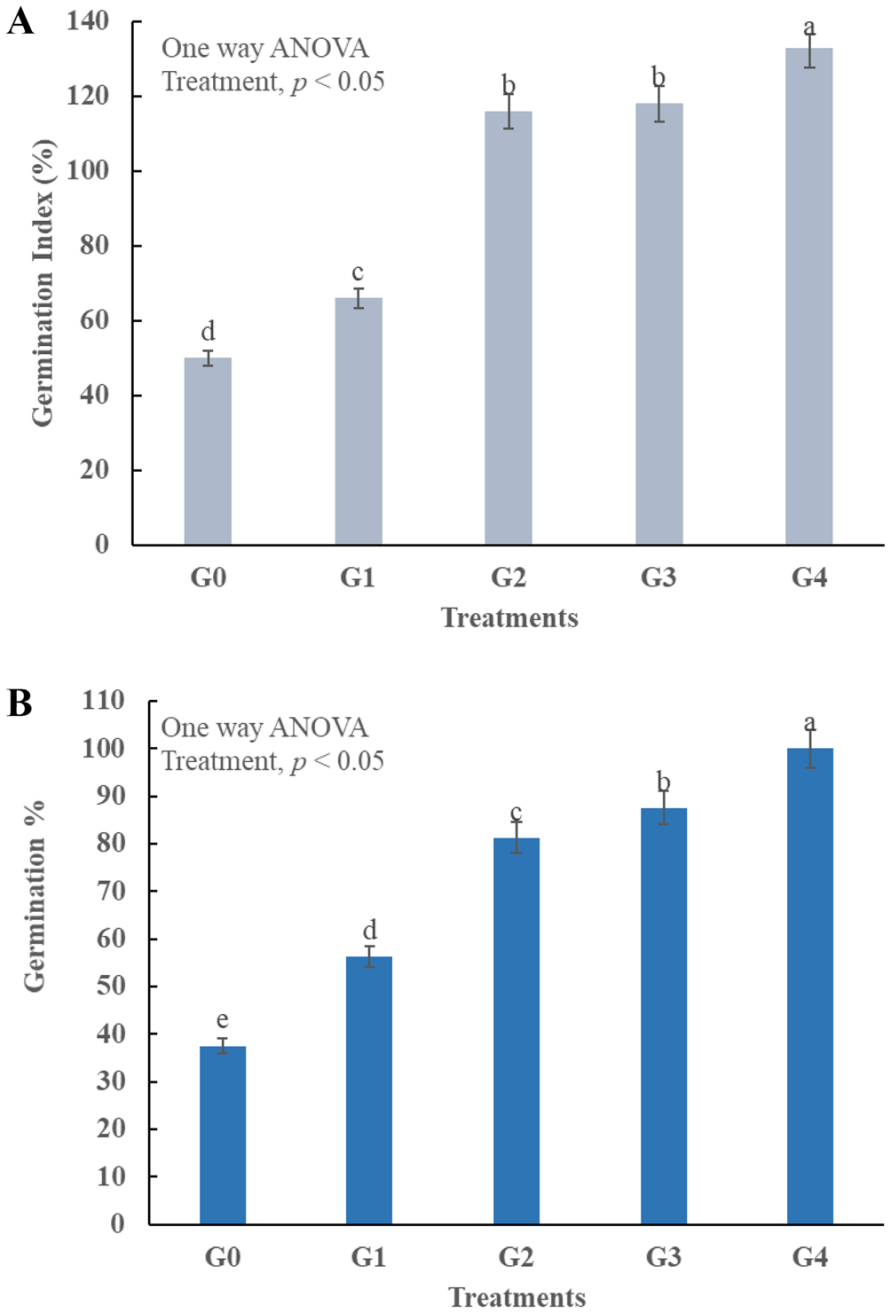
Impact of synthesized composts in germination experiment G0 = without compost, G1 = compost of kitchen waste, G2 = compost of Kitchen water + tea waste, G3 = compost of Kitchen waste + biochar, G4 = compost of Kitchen waste + tea waste + biochar Lignin degradation, (**A**) compost influence on germination index %, (**B**) compost influence on Germination %.

Phytotoxicity determination is a crucial parameter to check the compost maturity^53^. The present study, has performed different experiments to check whether the compost is matured enough or still contain phytotoxic substances. Compost is considered mature when it has GI higher than 80%^3^. GI experiment was performed in four treatments and they were significantly (*p* < 0.05) different. G1 was 66% which indicates the presence of phytotoxic substances in the compost and it is not yet matured (Fig. 6A, Table 2). GI of other three treatments is in the range of 116–133%, this is due to the combined adsorption properties of TW and BC. Bulking agents also provide adsorption of volatile^33^ and toxic substances^50^. G% indicates the germination rate of the compost and G_4_ has achieved germination rate of 100% that shows all the seeds have grown (Fig. 6B). This is because that TW has the ability to produce humic substances^14^, which make root cell membrane structurally more stable^17^. Humic substances also make cell more permeable to take up nutrients^15^ and resultantly enhance the plant growth^54^. The mechanism for BC is different, it has high water retention potential that results in slow mineralization^55^ and less leachate production^53^. This results in reduction in nutrient loss^43^ and consequently increase the plant growth^46^.

### Seed vigor index and Munoo-Liisa vitality index

**Figure 7:**
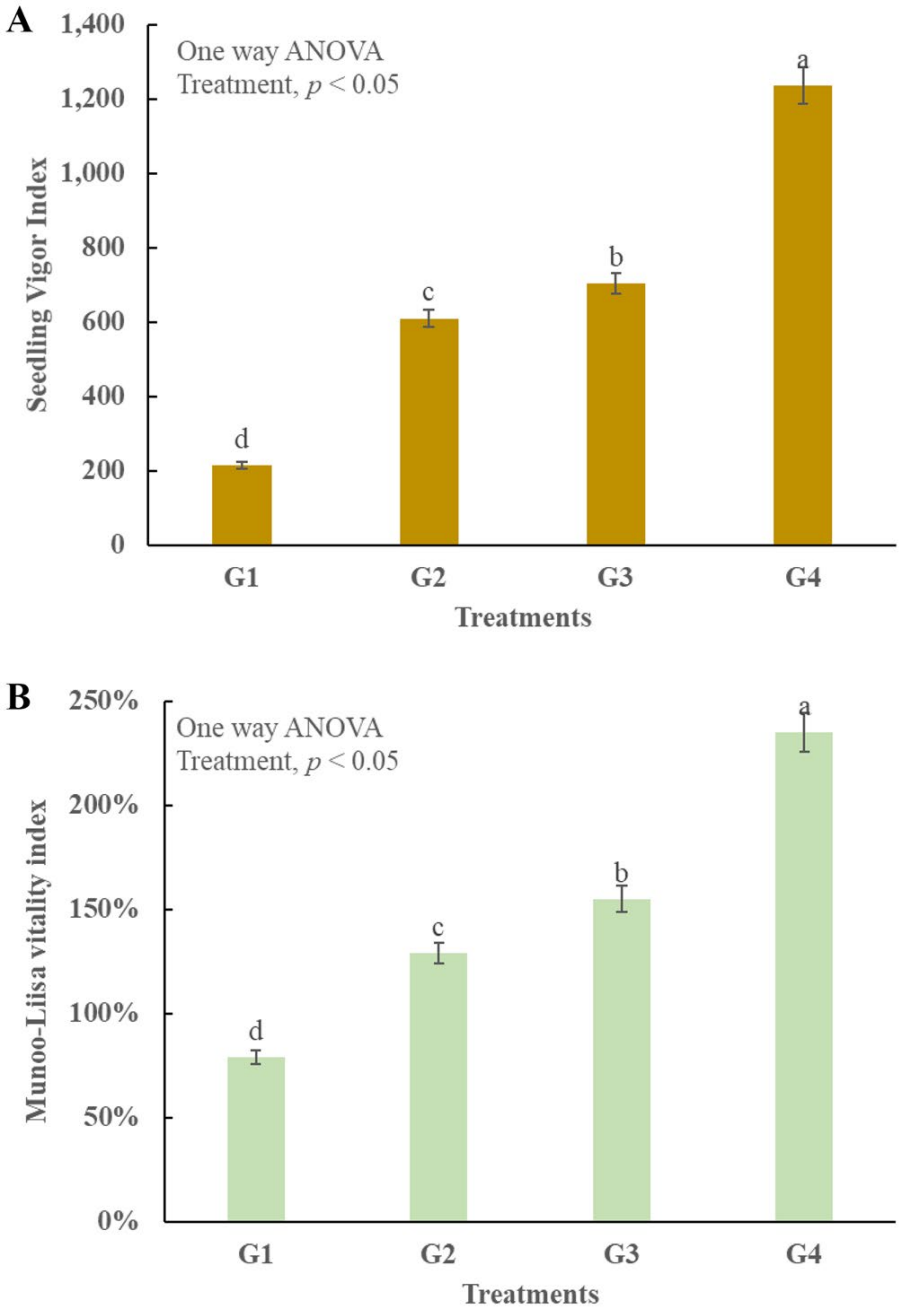
Impact of synthesized composts on seed vigor and vitality, G1 = compost of kitchen waste, G2 = compost of Kitchen water + tea waste, G3 = compost of Kitchen waste + biochar, G4 = compost of Kitchen waste + tea waste + biochar Lignin degradation, (**A**) compost influence on germination vigor index, (**B**) compost influence on Munoo-Liisa vitality index.

Seed vigor index (SVI) shows the ability of the seeds to grow in the provided conditions, it is directly proportional to the crop yield^55^. The present study reported *p* < 0.05, which means that all treatments have different capability to facilitate. G_4_ showed highest SVI of 1238 and the lowest vigor values are found in G_1_ (Fig. 7A). High SVI is also linked with high phosphorus content^29^ and nutrient availability^32^. Likewise, high porosity and high WHC of compost facilitate the seed growth^27^ and results in high SVI^40^. Munoo-Liisa Vitality Index (MVI) in an important indicator that shows how much crop yield can be produced using a specific amendment^56^. The standard value of MVI is set at 80% by different agricultural departments, internationally^26^. In this study, G_1_ did not match up to the set target while G_4_ showed the highest yield percentage (Fig. 7B, Table 3). Values lower than 80% are indicative of the fact there are potential phytotoxic substances present in the compost^54^.

### Practical implications and future prospect

This study has successfully established that biochar and tea waste can be used in kitchen waste composting as it is not harmful along with its milk content. This study will help promote organic farming as the compost is cheaper and have the high nutritional value and can replace chemical fertilizers that are expensive and are harmful for long term use (Table 4). Secondly, most of the farmers and commercial composting plants does not use kitchen waste that is mixed with tea waste because of milk that has the non-vegetarian origin. Kitchen waste co-compost can be an economical method for reducing food security (Table 4). Kitchen waste compost maturity is a serious challenge and as per the findings of this study it can be best dealt with using biochar. It has a good capacity for water adsorption and consequently generating pressure which is good for heating up the composting system. It has been established that composting with bulking agents and raw material is a more sustainable option than incineration and open dumping. Rather throwing kitchen waste, we can easily convert this into a useful resource (compost). In developing countries, low income groups can use this compost to grow vegetables in home/kitchen gardening. This can be helpful to overcome supply shortage and food inflation.

**Table 4:** Comparison table of studies on different compost types.

Raw material	Compost bulking agent	Parameters	Results	Maturation time	Reference
Kitchen waste	Dry leaves, tree pruning	C/N	11–16	28 days	Li et al.^2^
		GI %	83–120		
Kitchen waste	Biochar, Tea waste	Temp °C	36–37	30 days	This study
		MC %	15–33		
		pH	7.0–8.5		
		EC mS/cm	1.5–7		
		C/N	14–22		
		OM %	41–60		
		GI %	83–133		
Food waste	Maize stalk	OM%	10	30 days	Ahmed et al.^17^
Green waste	Bean dregs, tea waste, biochar	OM	28–58	30 days	Li et al.^3^
		C/N	8–11		
		GI	80–173		
Vegetable waste	Garden prune, Saw dust	pH	6–8	30 days	Rich et al.^4^
		Temperature	40–55		
		C/N	23–13		
Saw dust	Wood chips	OM %	39–47	32 days	Nguyen et al.^18^
		EC mS/cm	2.5–1.7		
		C/N	13–14.8		
Municipal sewage sludge	Rapseed straw	DM	35	35 days	Petrovic et al.^56^
		pH	7.3–7.9		
		Temp °C	23–30		
		Oxygen	20–20.5		
Kitchen waste	Cornstalk	C/N	11–13	35 days	Yang et al.^5^
		CH4	80–85		
		NH3	18–23		
		GI %	38–122		
Pig manure	Wheat straw	OM %	47–51	49 days	Yang et al.^6^
		EC mS/cm	1.5–1.7		
		C/N	11–11.8		
		DOC g/kg	7–9		
Poultry litter	Rice husk	OM	50	65 days	Alarefee et al.^45^
Chicken manure	Sawdust	pH	7.4–7.6	115 days	Gao et al.^9^
		EC µS/cm	2050–2600		
		GI %	60–94		
		NH4^+^ N mg/kg	350–1073		

### Recommendations

- Tea waste should be incorporated in kitchen waste composting in order to minimize the amount of solid waste produced.
- Application of compost to soil and its impact on plant growth and soil quality for a longer period of times should be studied.
- Composting of kitchen waste is an inexpensive method compared to the chemical fertilizer application.
- More studies should be carried out in open fields with different variables and optimization techniques.
- The findings of this study will help promote kitchen waste composting with tea residue without producing harmful impact.
- The time barrier for kitchen waste composting has been reduced using tea waste (another waste). So, this study provides a feasible option to recycle both kitchen and tea waste effectively.

## Conclusion

The present study showed that tea waste is not harmful along with milk contents, it can be used in composting of kitchen waste. The study showed that teas waste and biochar are effective bulking agents and created positive effect on maturation of kitchen waste compost. They improved the compost quality by enhancing organic matter, lignocellulose degradation, water holding capacity, porosity and plant growth. Compost get matured in 30 days and can be used as organic fertilizer. The combined addition of both bulking agents enhanced the growth of tomato plant. This study successfully met its objectives and recommended a practical method for accelerating kitchen waste composting. Further studies must be carried out to study the long-term impact of organic compost on plants. Long maturation time is a serious challenge in kitchen waste composting and this study effectively reduced the maturation time by using biochar. Composting with bulking agents and raw material is more sustainable option than incineration and open dumping. The results of this research can be used for home composting and successively in home/kitchen gardening. This can be beneficial in reducing the municipal solid waste, reducing burden on landfill sites, converting waste into a resource. In developing countries this home composting and home gardening can also help to overcome food security issues.

## Data Availability

The datasets generated during and/or analyzed during the current study are available from the corresponding author on reasonable request.

## Abbreviations

BC: Biochar
BD: Bulk density
C/N: Carbon nitrogen ratio
EC: Electrical conductivity
G%: Germination percentage
GHG: Greenhouse gas
GI: Germination Index
KW: Kitchen waste
LOI: Loss on ignition
MC: Moisture content
MVI: Munoo-Liisa vitality index
OM: Organic matter
SVI: Seedling vigor index
TC: Total carbon
TK: Total potassium
TN: Total nitrogen
TP: Total phosphorus
TW: Tea waste
WHC: Water holding capacity
